# Reliability Assessment of Harmonic Reducers Based on the Two-Phase Hybrid Stochastic Degradation Process

**DOI:** 10.3390/s26082437

**Published:** 2026-04-15

**Authors:** Lai Wei, Peng Liu, Hailong Tian, Haoyuan Li, Yunshenghao Qiu

**Affiliations:** 1 Key Laboratory of CNC Equipment Reliability, Ministry of Education, Jilin University, Changchun 130022, China; laiweidavid@163.com (L.W.); liupeng@jlu.edu.cn (P.L.); lhaoy99@163.com (H.L.); yunshenghao.qiu@uqconnect.edu.au (Y.Q.); 2 College of Mechanical and Aerospace Engineering, Jilin University, Changchun 130022, China

**Keywords:** harmonic reducer, hybrid stochastic degradation process, two-phase, change point, reliability assessment

## Abstract

Harmonic reducers exhibit non-stationary and phase-dependent degradation behavior during long-term service, challenging the ability of classical stochastic degradation models to accurately assess reliability. To address phase-dependent differences in degradation behavior, this paper proposes a reliability assessment model based on a two-phase hybrid stochastic degradation process. In the proposed framework, the Wiener process is employed to characterize early-phase gradual degradation dominated by stochastic fluctuations, while the Inverse Gaussian process is used to describe later-phase monotonically accelerated degradation driven by cumulative damage. The framework allows for sample-level variability in transition times to more realistically capture individual degradation behavior. The Schwarz Information Criterion is also adopted to detect change points. Maximum likelihood estimation is performed for model parameter inference, and analytical expressions for the reliability function, cumulative distribution function, and probability density function are derived. Numerical results indicate that a change point exists for each tested product and that the proposed model achieves the best goodness of fit among the considered candidates, demonstrating its superiority in capturing phase-dependent characteristics of harmonic reducer degradation. In terms of reliability assessment bias, the proposed model (0.06%) significantly outperforms the Wiener degradation model (32%) and the IG degradation model (9.9%). These results further confirm that, under an identical failure threshold, the proposed approach yields more accurate and realistic reliability assessment outcomes.

## 1. Introduction

With the rapid development of precision manufacturing and sensing technologies, harmonic reducers have become key functional components in industrial robots, high-end CNC machine tools, and aerospace equipment given their compact size, light weight, high precision, and high transmission efficiency. Their continuously expanding application scale imposes increasingly stringent requirements on reliability performance [1]. Influenced by factors such as gear meshing wear, load fluctuations, and variations in lubrication conditions, harmonic reducers typically exhibit a two-phase degradation pattern during long-term service, transitioning from a steady degradation phase to an accelerated one. In the early phase, degradation is dominated by relatively small random fluctuations due to the protective isolation provided by lubricants and the elastic buffering effects of materials, resulting in largely reversible or slow degradation. As service time extends and internal damage accumulates, the degradation rate accelerates in the later phase, accompanied by enhanced monotonicity. Therefore, key performance indicators, such as transmission efficiency, vibration level, and transmission accuracy, exhibit pronounced cumulative damage [2,3,4,5]. Given this phase-dependent degradation behavior, two-phase stochastic modeling is typically adopted. As the early degradation phase is characterized by a relatively gradual degradation process accompanied by random fluctuations, it is usually modeled as a Wiener process, whereas the later accelerated and monotonic degradation phase driven by cumulative damage tends to be described as an Inverse Gaussian (IG) process.

To gain deeper insights into system failure mechanisms, reliability engineering increasingly relies on degradation modeling and analysis as a core methodological tool [6,7]. Moreover, degradation modeling facilitates more accurate characterization of complex degradation behaviors and improves the precision of reliability assessment [8]. Accordingly, various extensions of the Wiener process have been proposed, including models that account for individual heterogeneity and measurement errors [9] and models incorporating random initial values [10]. For example, Zhai, Li, and Chen [11] modeled the drift parameters of the Wiener process using random effects and further derived the corresponding reliability and probability density functions.

Recent years have seen an increase in reliability modeling research in systems with multi-phase degradation characteristics [12]. These systems are typically characterized by phase-dependent degradation behavior influenced by two factors. (1) Phased changes have various environmental causes, prompting the need for artificial phase division during reliability analysis. For example, semiconductor lasers operate under different driving currents and operating temperatures [13]. (2) Phased changes are determined by the specific time or space of the task, e.g., military armored vehicles perform marching and combat missions under different terrain conditions [14]. 

As such, degradation modeling research has advanced to more accurately characterize multi-phase degradation behavior, including traditional stochastic degradation processes, multi-phase stochastic degradation processes, and hybrid stochastic degradation processes. Traditional stochastic degradation processes, including the Wiener, Gamma, and Inverse Gaussian (IG) processes, are commonly employed to characterize product degradation behavior. The literature has predominantly focused on the Wiener process [15,16] and the Gamma process [17,18] to model wear accumulation, as well as the IG process for fatigue crack propagation [19,20]. Zhang et al. [21] broadened the application scope of this paradigm by introducing a branching process to construct a stochastic multi-phase model. Ma et al. [22] improved the multi-phase Wiener process model based on the impact of imperfect equipment maintenance, while Zhang et al. [23] constructed a general degradation modeling framework based on the two-phase Wiener process and proposed a new lifetime estimation method. Furthermore, Gao, Cui, and Dong [24] and Liang et al. [25] demonstrated that multi-phase degradation processes based on a two-phase Wiener process and a two-phase IG process exhibit continuity and interdependence between phases while maintaining overall coherence under a unified distribution framework.

These multi-phase degradation process models remain within the framework of stochastic degradation processes, and differences between phases are primarily represented by phase-dependent parameters, limiting their ability to capture the mixed stochastic characteristics of degradation behavior. In contrast, from a theoretical perspective, hybrid stochastic degradation processes integrate different stochastic characteristics within the same degradation phase while representing parameter variations through time-based segmentation. Consequently, in practical engineering applications, the introduction of hybrid stochastic degradation processes enables a more realistic representation of product degradation behavior.

Change point modeling has emerged as a way to characterize phase-dependent structural changes in degradation trajectories, attracting considerable attention in reliability analysis in recent years. Kong and Cui [26] first introduced the concept of the change point and developed a nonlinear two-phase Wiener degradation process model for reliability assessment. Subsequently, they extended this two-phase modeling framework to a multi-phase setting by constructing a multi-phase Wiener process model and employing Bayesian inference for reliability assessment [27]. Building on this work, Lin et al. [28] proposed an improved multi-phase Wiener process model that explicitly accounts for variability in change points and parameter heterogeneity among different systems. In parallel, Zhuang et al. [29] adopted a two-phase reparameterized IG process model to more accurately capture the degradation behavior of complex systems and enhance reliability assessment accuracy. Gu et al. [30] introduced a change-point-based segmentation strategy for the IG process, established a two-phase degradation model, and derived the corresponding reliability expressions, providing an effective framework for modeling phased degradation behavior. Beyond system-level reliability assessment, change point modeling has also been widely applied to related research topics, including remaining useful life prediction [31,32], multi-sensor degradation modeling [33,34], and degradation process simulation techniques [35,36].

Despite the significant progress achieved in change point degradation modeling and successful applications across various fields, several challenges remain for harmonic reducers.

(1) Although harmonic reducers have attracted increasing attention in reliability research, studies specifically devoted to harmonic reducers are limited. Most existing work focuses more on failure analysis [3,4], performance variation description [2,37], or simulation-based investigation [38,39] rather than probabilistic degradation modeling based on laboratory degradation test data. Therefore, the corresponding degradation characteristics of harmonic reducers may not yet be sufficiently captured from a reliability assessment perspective.

(2) Most existing multi-phase degradation models are developed within the same stochastic process family, where phase differences are represented by parameter changes. This modeling strategy may be inadequate for characterizing the mixed stochastic properties of harmonic reducer degradation across different phases.

(3) Existing change point degradation models usually assume that all tested products share an identical transition time between degradation phases without explicitly considering possible heterogeneity in the transition behavior of individual units. However, under practical operating conditions, different harmonic reducers may exhibit distinct transition times due to individual differences and accumulated damage variability.

To address these challenges, this paper proposes a reliability assessment method based on a two-phase hybrid stochastic degradation process. The main contributions of the paper are summarized as follows.

(1) A two-phase hybrid stochastic degradation process model is developed based on the Schwarz Information Criterion (SIC) for change point detection and model selection. The proposed model couples the nonlinear Wiener process with the nonlinear IG process to characterize the potential change point in the degradation trajectory of harmonic reducers, where the Wiener process describes the gradual degradation phase prior to the change point and the IG process captures the accelerated degradation phase thereafter.

(2) Unlike existing studies, this work develops a framework that allows for sample-level variability in transition times in the parameter estimation framework, enabling more accurate characterization of individual products’ degradation behavior. In addition, the concept of first passage time is introduced, and expressions for the reliability function, cumulative distribution function (CDF), and probability density function (PDF) of the proposed two-phase model are derived.

(3) The proposed model is validated using a numerical example of the performance degradation of a harmonic reducer. The results demonstrate that the two-phase hybrid stochastic degradation process more accurately captures the phase-dependent characteristics of harmonic reducer degradation compared to traditional stochastic degradation models. This supports more accurate reliability assessment and life analysis of two-phase hybrid stochastic degradation processes.

The remainder of this paper is organized as follows. Section 2 introduces the theoretical framework of the SIC and formulates hypothesis testing for the candidate models. Hypothesis testing is based on the non-change point and the single-change point. Based on this discriminant framework, the Wiener and the IG process models, together with a two-phase hybrid stochastic degradation process model that integrates the two traditional models, are developed. Section 3 presents the procedures for estimating the unknown model parameters. Section 4 derives the reliability function, cumulative distribution function CDF, and PDF for each model. Section 5 provides a numerical example based on harmonic reducer degradation data to demonstrate the effectiveness of the proposed model and method. Section 6 summarizes the results.

## 2. Model Description

### 2.1. Schwartz Information Criterion

Schwartz proposed the SIC in 1978, which can be applied to model selection problems [40]. The principle is that when there is a change point in the data being analyzed, the SIC value of the sample will be greater than the SIC value of a sample without the change point. The expression is shown in Equation (1):

(1) $$SIC\left(m\right)=-2\ln L+V\ln m$$

where lnL is the log-likelihood function of the model, V is the number of model parameters, and m is the sample size.

Based on the judgment of the presence or absence of a change point, hypothesis testing for the candidate models is formulated in terms of the non-change hypothesis ($H_{0}$) and the change point hypothesis ($H_{1}$).

### 2.2. Wiener and IG Degradation Process Model Description

First, a Wiener process model is established to represent the continuous performance degradation process, as shown in Equation (2):

(2) $$X^{Wiener}(t)=X(0)+\eta t+\sigma B(t)$$

where $X^{Wiener}(t)$ follows the normal distribution, X(0)=0 is the initial performance degradation value, η is the drift parameter, σ is the diffusion parameter, and B(t) is the standard Wiener degradation process.

Second, an IG process model with independent and non-negative increments is established to describe the strictly monotonic performance degradation process, as shown in Equation (3):

(3) $$X^{IG}(t)\sim IG\left(\mu t,{\lambda t}^{2}\right)$$

where $X^{IG}(t)$ follows the IG distribution, μ is the mean parameter, and λ is the shape parameter.

Based on this, incremental Wiener and IG degradation process models should be considered, as shown in Equations (4) and (5):

(4) $$H_{0}^{Wiener}:\Delta x_{Q}\sim N\left(\eta \Delta t_{Q},\sigma^{2}\Delta t_{Q}\right)$$

(5) $$H_{0}^{IG}:\Delta x_{Q}\sim IG\left(\mu \Delta t_{Q},\lambda \Delta {t_{Q}}^{2}\right)$$

The SIC expressions for the two non-change point hypotheses are shown in Equations (6) and (7), respectively:

(6) $$H_{0}^{Wiener}:{SIC}_{0}^{Wiener}\left(m\right)=-2\ln L^{Wiener}+V^{Wiener}\ln m$$

(7) $$H_{0}^{IG}:{SIC}_{0}^{IG}\left(m\right)=-2\ln L^{IG}+V^{IG}\ln m$$

where $V^{Wiener}=V^{IG}=2$. The minimum of the two values is taken as the overall evaluation value of the non-change point hypothesis, as shown in Equation (8):

(8) $$H_{0}=\left\{H_{0}^{Wiener},H_{0}^{IG}\right\}:{SIC}_{0}\left(m\right)=min\left\{{SIC}_{0}^{Wiener}\left(m\right),{SIC}_{0}^{IG}\left(m\right)\right\}$$

### 2.3. Two-Phase Hybrid Stochastic Degradation Process Model Description

It is assumed that the degradation process of the system is governed by the Wiener process before the change point and by the IG process thereafter, forming a two-phase hybrid stochastic degradation process model. At any phase, once product performance reaches a threshold, it is considered a failure.

According to the properties of the Wiener degradation process model, the increment $X\left(t,t+\Delta t\right)-X\left(t\right)\sim N\left(\eta \Delta t,\sigma^{2}\Delta t\right)$ on any interval $\left[t,t+\Delta t\right]$. As such, the Wiener process can model non-monotonic degradation processes. Simultaneously, if the influence of external conditions on product performance degradation is relatively weak, i.e., $\frac{\eta }{\sigma^{2}}\geq 1$, a situation where the degradation increment is negative can be ignored. The degradation process can be approximated as a monotonically decreasing process. Both monotonic and non-monotonic processes can be modeled using the Wiener degradation process.

According to the degradation test, the total number of tests conducted on the product within time t is M+1 degradation data points $x_{M}$, where M=0,1,2,⋯,m. As both the Wiener degradation process and the IG degradation process have independent incremental characteristics, the increment is calculated for the data in each phase, assuming the time of change is $t_{q}$. The first phase is the Wiener degradation process, i.e., $X^{Wiener}(t|\eta ,\sigma^{2})$, which yields q performance degradation increment data points $\Delta x_{Q}=x\left(t_{Q}\right)-x\left(t_{Q-1}\right),\;Q=0,1,2,\cdots ,q$. The second phase is the IG degradation process, i.e., $X^{IG}(t|\mu ,\lambda )$, which yields m−q performance degradation increment data points $\Delta x_{Q}=x\left(t_{Q}\right)-x\left(t_{Q-1}\right),\;Q=q+1,\cdots ,m$.

The expression for the two-phase hybrid stochastic degradation process model is shown in Equation (9):

(9) $$X(t)=\left\{\begin{array}{c} X^{Wiener}(t)\sim N\left(\eta t,\sigma^{2}t\right),\;0\;{\leq t\leq t}_{q}\\ X^{IG}(t)\sim IG\left(\mu t,{\lambda t}^{2}\right),\;t_{q}<t \end{array}\right.$$

The performance degradation increment in the first phase of the sample follows a normal distribution, while the performance degradation increment in the second phase follows an IG distribution, as shown in Equation (10):

(10) $$\left\{\begin{array}{c} \Delta x_{q}\sim N\left(\eta \Delta t_{q},\sigma^{2}\Delta t_{q}\right)\\ \Delta x_{q+1}\sim IG\left(\mu \Delta t_{q+1},\lambda {\left(\Delta t_{q+1}\right)}^{2}\right) \end{array}\right.$$

The SIC expressions for this model are shown in Equation (11):

(11) $$H_{1,t_{q}}:{SIC}_{1}\left(q\right)=-2\left[\ln L^{Wiener}\left(\eta ,\sigma^{2}\right)+\ln L^{IG}\left(\mu ,\lambda \right)\right]+V_{1}\ln m$$

where $V_{1}=4$. The hypothesis assumes a steady stochastic degradation process before the change point, which subsequently transitions into a cumulative degradation process.

$H_{0}$ is accepted when ${SIC}_{0}\left(m\right)\leq \underset{q\in \left[2,m-2\right]}{\min}{SIC}_{1}\left(q\right)$, indicating that the sequence as a whole follows the same distribution and without the change point; if there exists certain $q\left(q\in \left[2,m-2\right]\right)$ such that ${SIC}_{0}\left(m\right)>\underset{q\in \left[2,m-2\right]}{\min}{SIC}_{1}\left(q\right)$, then $H_{0}$ is rejected, while the alternative hypothesis $H_{1,t_{q}}$ is accepted, indicating that there is a change point q in the degraded data $x_{1},x_{2}{,\cdots ,x}_{M}$. We define the change point location as $\hat{q}$ and use $\hat{q}$ to estimate the specific location of the change point q. The judgment condition is shown in Equation (12):

(12) $${SIC}_{1}\left(\hat{q}\right)=\underset{q\in \left[2,m-2\right]}{\min}{SIC}_{1}\left(q\right)$$

Among all candidate change points, the one corresponding to the minimum SIC value is selected as the estimated change point location, as shown in Equation (13):

(13) $$\hat{q}=arg\underset{q\in \left[2,m-2\right]}{\min}{SIC}_{1}\left(q\right)$$

This procedure is repeated for all samples to obtain the estimated change point locations $\hat{q}=\left\{{\hat{q}}_{1},{\hat{q}}_{2},\cdots ,{\hat{q}}_{n}\right\}$ for each sample. When the estimated change point locations show strong consistency across samples and do not exhibit obvious dispersion or grouping characteristics, this common location can be treated as a unified change point for all tested harmonic reducers, and a two-phase hybrid stochastic degradation process model is constructed based on this unified change point for overall parameter estimation. Conversely, if substantial variability exists among the sample-specific change point estimates, ${\hat{q}}_{1},{\hat{q}}_{2},\cdots ,{\hat{q}}_{n}$ are retained, and the sample-level differences are regarded as manifestations of randomness and heterogeneity in the degradation behavior.

## 3. Parameter Estimation

### 3.1. Estimation of Wiener and IG Process Models

According to Section 2.2, the parameters to be estimated are $\Theta^{Wiener}=\left(\eta ,\sigma^{2}\right),\Theta^{IG}=\left(\mu ,\lambda \right)$. There is a total of $N\left(N=1,2,\cdots ,n\right)$ harmonic reducer samples in the experiment. The measured value of the N-th sample is $x_{N}=\left(x_{N,0}{,x}_{N,1},\cdots ,x_{N,M_{n}}\right)$. $\Delta x_{N,M}=x_{N,M}-x_{N,M-1}$ represents the increment of performance degradation and $t_{N,M}$ represents the M-th observation time of the N-th sample, where $M=1,2,\cdots ,m_{n}$.

The parameters corresponding to the N-th sample data are $\Theta_{N}^{Wiener}=\left(\eta_{N},\sigma_{N}^{2}\right),\Theta_{N}^{IG}=\left(\mu_{N},\lambda_{N}\right)$. The log-likelihood function expressions for the N-th sample information, ${\ln L}_{N}^{Wiener}$ and ${\ln L}_{N}^{IG}$, are shown in Equations (14) and (15):

(14) $${\ln L}^{Wiener}\left(\Delta x_{N,M}|\Theta_{N}^{Wiener}\right)=\sum_{M=1}^{m_{n}}\left[-\frac{1}{2}\ln\left(2\pi \sigma_{N}^{2}\Delta t_{N,M}\right)-\frac{{\left(\Delta x_{N,M}-\eta_{N}\Delta t_{N,M}\right)}^{2}}{2\sigma_{N}^{2}\Delta t_{N,M}}\right]$$

$$\ln L^{IG}\left(\Delta x_{N,M}|\Theta_{N}^{IG}\right)$$

(15) $$=\sum_{M=1}^{m_{n}}\begin{array}{c} \left[\begin{array}{c} \frac{1}{2}\ln\lambda_{N}+\ln\left(\Delta t_{N,M}\right)-\frac{1}{2}\ln\left(2\pi \right)\\ -\frac{3}{2}\ln\left(\Delta x_{N,M}\right)-\frac{\lambda_{N}{\left(\Delta x_{N,M}-\mu_{N}\Delta t_{N,M}\right)}^{2}}{2{\left(\mu_{N}\right)}^{2}\Delta x_{N,M}} \end{array}\right] \end{array}$$

Taking the first-order partial derivatives of $\Theta_{N}^{Wiener}$ and $\Theta_{N}^{IG}$ and setting them equal to 0, we can obtain the closed-form solutions as shown in Equations (16) and (17):

(16) $${\hat{\Theta }}_{N}^{Wiener}=\left\{\begin{array}{c} \begin{array}{c} {\hat{\eta }}_{N}=\sum_{M=1}^{m_{n}}\frac{\Delta x_{N,M}}{\Delta t_{N,M}}\\ {\hat{\sigma }}_{N}^{2}=\frac{1}{m_{n}}\sum_{M=1}^{m_{n}}\frac{{\left(\Delta x_{N,M}-{\hat{\eta }}_{N}\Delta t_{N,M}\right)}^{2}}{\Delta t_{N,M}} \end{array} \end{array}\right.$$

(17) $${\hat{\Theta }}_{N}^{IG}=\left\{\begin{array}{c} {\hat{\mu }}_{N}=\sum_{M=1}^{m_{n}}\frac{\Delta x_{N,M}}{\Delta t_{N,M}}\\ {\hat{\lambda }}_{N}=\frac{m_{n}}{\sum_{M=1}^{m_{n}}\frac{{\left(\Delta x_{N,M}-{\hat{\mu }}_{N}\Delta t_{N,M}\right)}^{2}}{{\left({\hat{\mu }}_{N}\right)}^{2}\Delta x_{N,M}}} \end{array}\right.$$

Following this, log-likelihood functions ${\ln L}^{Wiener}$ and $\ln L^{IG}$ containing all sample information are constructed, as shown in Equations (18) and (19):

(18) $${\ln L}^{Wiener}\left(\Delta x_{N,M}|\Theta^{Wiener}\right)=\sum_{N=1}^{n}\sum_{M=1}^{m_{n}}\left[-\frac{1}{2}\ln\left(2\pi \sigma^{2}\Delta t_{N,M}\right)-\frac{{\left(\Delta x_{N,M}-\eta \Delta t_{N,M}\right)}^{2}}{2\sigma^{2}\Delta t_{N,M}}\right]$$

$$\ln L^{IG}\left(\Delta x_{N,M}|\Theta^{IG}\right)$$

(19) $$=\sum_{N=1}^{n}\sum_{M=1}^{m_{n}}\begin{array}{c} \left[\begin{array}{c} \frac{1}{2}\ln\lambda +\ln\left(\Delta t_{N,M}\right)-\frac{1}{2}\ln\left(2\pi \right)\\ -\frac{3}{2}\ln\left(\Delta x_{N,M}\right)-\frac{\lambda {\left(\Delta x_{N,M}-\mu \Delta t_{N,M}\right)}^{2}}{2\mu^{2}\Delta x_{N,M}} \end{array}\right] \end{array}$$

Taking the first-order partial derivatives of $\Theta^{Wiener}$ and $\Theta^{IG}$ and setting them equal to 0, we can obtain the closed-form solutions as shown in Equations (20) and (21):

(20) $${\hat{\Theta }}^{Wiener}=\left\{\begin{array}{c} \begin{array}{c} \hat{\eta }=\sum_{N=1}^{n}\sum_{M=1}^{m_{n}}\frac{\Delta x_{N,M}}{\Delta t_{N,M}}\\ {\hat{\sigma }}^{2}=\frac{1}{\sum_{N=1}^{n}m_{n}}\sum_{N=1}^{n}\sum_{M=1}^{m_{n}}\frac{{\left(\Delta x_{N,M}-\hat{\eta }\Delta t_{N,M}\right)}^{2}}{\Delta t_{N,M}} \end{array} \end{array}\right.$$

(21) $${\hat{\Theta }}^{IG}=\left\{\begin{array}{c} \hat{\mu }=\sum_{N=1}^{n}\sum_{M=1}^{m_{n}}\frac{\Delta x_{N,M}}{\Delta t_{N,M}}\\ \hat{\lambda }=\frac{\sum_{N=1}^{n}m_{n}}{\sum_{N=1}^{n}\sum_{M=1}^{m_{N}}\frac{{\left(\Delta x_{N,M}-\hat{\mu }\Delta t_{N,M}\right)}^{2}}{{\hat{\mu }}^{2}\Delta x_{N,M}}} \end{array}\right.$$

### 3.2. Estimation of Two-Phase Hybrid Stochastic Process Model

It is essential to explain how the change point time $t_{q}$ is handled before performing parameter estimation on the two-phase hybrid stochastic process model. $t_{q}$ is not used as a parameter to be estimated in the maximum likelihood estimation (MLE) algorithm, but it is pre-identified at the sample level through SIC (see Equation (13)). The corresponding change point time ${\hat{t}}_{N,q_{n}}$ is solved for each test sample separately and treated as a known constant in the subsequent parameter estimation. Hence, change point identification is separated from parameter estimation so that the change point position and model parameters do not need to be jointly optimized in the MLE process, which does not introduce a non-smooth log-likelihood structure and ensures the convergence stability of the estimation results.

According to Section 2.3, the parameters to be estimated are $\Theta_{Two\text{-}phase}^{*}=\left(\eta^{*},{\left(\sigma^{2}\right)}^{*},\mu^{*},\lambda^{*}\right)$. The prerequisite for estimating $\Theta_{Two\text{-}phase}^{*}$ is to use SIC to determine whether there exists a change point for each data sample. If it exists, the selection interval is constructed for the change point of the Nth data sample, and the time interval before and after the change point is divided into $\Delta \tau_{1}\left(t_{N,M}\right)$ and $\Delta \tau_{2}\left(t_{N,M}\right)$, as shown in Equation (22):

(22) $$Z_{n}=\left\{\left[\tau_{1}\left(t_{N,q_{n}}\right),\tau_{2}\left(t_{N,q_{n}+1}\right)\right],\;q_{n}=\left(2,\cdots ,m_{n}-2\right)\right\}$$

The parameters corresponding to the Nth data sample are $\Theta_{Two\text{-}phaseN}^{*}=\left(\eta_{N}^{*},{\left(\sigma_{N}^{2}\right)}^{*},\mu_{N}^{*},\lambda_{N}^{*}\right)$. The expression of the log-likelihood function ${\ln L}_{Two\text{-}phaseN}^{*}$ of the Nth sample information is as shown in Equation (23):

$$\ln L_{Two\text{-}phaseN}^{*}\left(\Delta x_{N,M}|{\hat{q}}_{n};\Theta_{Two\text{-}phaseN}\right)$$

(23) $$=\ln L_{Two\text{-}phaseN}^{1*}\left(\Delta x_{N,M}|\eta_{N}^{*},{\left(\sigma_{N}^{2}\right)}^{*}\right)+\ln L_{Two\text{-}phaseN}^{2*}\left(\Delta x_{N,M}|\mu_{N}^{*},\lambda_{N}^{*}\right)\;=\sum_{M=1}^{q_{n}}\left[-\frac{1}{2}\ln\left(2\pi {\left(\sigma_{N}^{2}\right)}^{*}\Delta \tau_{1}\left(t_{N,M}\right)\right)-\frac{{\left(\Delta x_{N,M}-\eta_{N}^{*}\Delta \tau_{1}\left(t_{N,M}\right)\right)}^{2}}{2{\left(\sigma_{N}^{2}\right)}^{*}\Delta \tau_{1}\left(t_{N,M}\right)}\right]\;+\sum_{M=q_{n}+2}^{m_{n}}\left[\begin{array}{c} \frac{1}{2}\ln\lambda_{N}^{*}+\ln\left(\Delta \tau_{2}\left(t_{N,M}\right)\right)-\frac{1}{2}\ln\left(2\pi \right)\\ -\frac{3}{2}\ln\left(\Delta x_{N,M}\right)-\frac{\lambda_{N}^{*}{\left(\Delta x_{N,M}-\mu_{N}^{*}\Delta \tau_{2}\left(t_{N,M}\right)\right)}^{2}}{2{\left(\mu_{N}^{*}\right)}^{2}\Delta x_{N,M}} \end{array}\right]$$

where the log-likelihood functions for the first and second phases are shown in Equations (24) and (25), respectively:

(24) $$\ln L_{Two\text{-}phaseN}^{1*}\left(\Delta x_{N,M}|\eta_{N}^{*},{\left(\sigma_{N}^{2}\right)}^{*}\right)=\sum_{M=1}^{q_{n}}\begin{array}{c} \left[-\frac{1}{2}\ln\begin{array}{c} \left(2\pi {\left(\sigma_{N}^{2}\right)}^{*}\Delta \tau_{1}\left(t_{N,M}\right)\right)\\ -\frac{{\left(\Delta x_{N,M}-\eta_{N}^{*}\Delta \tau_{1}\left(t_{N,M}\right)\right)}^{2}}{2{\left(\sigma_{N}^{2}\right)}^{*}\Delta \tau_{1}\left(t_{N,M}\right)} \end{array}\right] \end{array}$$

(25) $$\ln L_{Two\text{-}phaseN}^{2*}\left(\Delta x_{N,M}|\mu_{N}^{*},\lambda_{N}^{*}\right)=\sum_{M=q_{n}+2}^{m_{n}}\begin{array}{c} \left[\begin{array}{c} \frac{1}{2}\ln\lambda_{N}^{*}+\ln\left(\Delta \tau_{2}\left(t_{N,M}\right)\right)-\frac{1}{2}\ln\left(2\pi \right)\\ -\frac{3}{2}\ln\left(\Delta x_{N,M}\right)-\frac{\lambda_{N}^{*}{\left(\Delta x_{N,M}-\mu_{N}^{*}\Delta \tau_{2}\left(t_{N,M}\right)\right)}^{2}}{2{\left(\mu_{N}^{*}\right)}^{2}\Delta x_{N,M}} \end{array}\right] \end{array}$$

Taking the first-order partial derivative with respect to $\Theta_{Two\text{-}phaseN}^{*}$ and setting it equal to 0, we can obtain the closed-form solutions, as shown in Equation (26):

(26) $${\hat{\Theta }}_{Two\text{-}phaseN}^{*}=\left\{\begin{array}{c} {\hat{\eta }}_{N}^{*}=\sum_{M=1}^{q_{n}}\frac{\Delta x_{N,M}}{\Delta \tau_{1}\left(t_{N,M}\right)}\\ \begin{array}{c} {\left({\hat{\sigma }}_{N}^{2}\right)}^{*}=\sum_{M=1}^{q_{n}}\frac{{\left(\Delta x_{N,M}-{\hat{\eta }}_{N}^{*}\Delta t_{N,M}\right)}^{2}}{\Delta \tau_{1}\left(t_{N,M}\right)} \end{array}\\ {\hat{\mu }}_{N}^{*}=\sum_{M=q_{n}+2}^{m_{n}}\frac{\Delta x_{N,M}}{\Delta \tau_{2}\left(t_{N,M}\right)}\\ {\hat{\lambda }}_{N}^{*}=\frac{m_{n}-q_{n}-1}{\sum_{M=q_{N}+2}^{m_{N}}\frac{{\left(\Delta x_{N,M}-{\hat{\mu }}_{N}^{*}\Delta \tau_{2}\left(t_{N,M}\right)\right)}^{2}}{{\left({\hat{\mu }}_{N}^{*}\right)}^{2}\Delta x_{N,M}}} \end{array}\right.$$

The change points $\hat{q}=\left({\hat{q}}_{1},{\hat{q}}_{2},\cdots ,{\hat{q}}_{n}\right)$ of each sample are solved according to the steps described in Section 2.3. Simultaneously, based on $\Theta_{Two\text{-}phase}^{*}=\left(\eta^{*},{\left(\sigma^{2}\right)}^{*},\mu^{*},\lambda^{*}\right)$, a log-likelihood function containing information from all samples is constructed, as shown in Equation (27):

$$\ln L_{Two\text{-}phase}^{*}\left(\Delta x_{N,M}|{\hat{q}}_{n};\Theta^{Wiener+IG}\right)$$

(27) $$=\sum_{N=1}^{n}\sum_{M=1}^{{\hat{q}}_{n}}\left[-\frac{1}{2}\ln\left(2\pi {\left(\sigma^{2}\right)}^{*}\Delta \tau_{1}\left(t_{N,M}\right)\right)-\frac{{\left(\Delta x_{N,M}-\eta^{*}\Delta \tau_{1}\left(t_{N,M}\right)\right)}^{2}}{2{\left(\sigma^{2}\right)}^{*}\Delta \tau_{1}\left(t_{N,M}\right)}\right]\;+\sum_{N=1}^{n}\sum_{M={\hat{q}}_{n}+2}^{m_{n}}\left[\begin{array}{c} \frac{1}{2}\ln\lambda^{*}+\ln\left(\Delta \tau_{2}\left(t_{N,M}\right)\right)-\frac{1}{2}\ln\left(2\pi \right)\\ -\frac{3}{2}\ln\left(\Delta x_{N,M}\right)-\frac{\lambda^{*}{\left(\Delta x_{N,M}-\mu^{*}\Delta \tau_{2}\left(t_{N,M}\right)\right)}^{2}}{2{\left(\mu^{*}\right)}^{2}\Delta x_{N,M}} \end{array}\right]$$

Taking the first-order partial derivatives of $\Theta_{Two\text{-}phase}^{*}$ and setting them equal to 0, we can obtain the closed-form solution, as shown in Equation (28):

(28) $${\hat{\Theta }}_{Two\text{-}phase}^{*}=\left\{\begin{array}{c} {\hat{\eta }}^{*}=\sum_{N=1}^{n}\sum_{M=1}^{{\hat{q}}_{n}}\frac{\Delta x_{N,M}}{\Delta \tau_{1}\left(t_{N,M}\right)}\\ {\left({\hat{\sigma }}^{2}\right)}^{*}=\frac{1}{\sum_{N=1}^{n}{\hat{q}}_{n}}\sum_{N=1}^{n}\sum_{M=1}^{{\hat{q}}_{n}}\frac{{\left(\Delta x_{N,M}-{\hat{\eta }}^{*}\Delta \tau_{1}\left(t_{N,M}\right)\right)}^{2}}{\Delta \tau_{1}\left(t_{N,M}\right)}\\ {\hat{\mu }}^{*}=\sum_{N=1}^{n}\sum_{M={\hat{q}}_{n}+2}^{m_{n}}\frac{\Delta x_{N,M}}{\Delta \tau_{2}\left(t_{N,M}\right)}\\ {\hat{\lambda }}^{*}=\frac{\sum_{N=1}^{n}m_{n}-{\hat{q}}_{n}-1}{\sum_{N=1}^{n}\sum_{M={\hat{q}}_{n}+2}^{m_{N}}\frac{{\left(\Delta x_{N,M}-{\hat{\mu }}^{*}\Delta \tau_{2}\left(t_{N,M}\right)\right)}^{2}}{{\left({\hat{\mu }}^{*}\right)}^{2}\Delta x_{N,M}}} \end{array}\right.$$

## 4. Reliability Function and Lifetime Distribution

The performance of harmonic reducers typically degrades over time during operation. A reliability function is defined based on the first time at which the degradation trajectory reaches a prescribed failure threshold, and the corresponding lifetime distribution is characterized using the CDF and the PDF. Accordingly, this section examines the Wiener process model, the IG process model, and the two-phase hybrid stochastic degradation process model and derives the associated expressions for the reliability function, CDF, and PDF. The differences in lifetime distributions arising from distinct degradation mechanisms are systematically revealed through this analysis.

### 4.1. Wiener Process Model

Assuming product lifetime is defined by first passage time (FPT) and denoted by a fixed failure threshold ω, the FPT of the product degradation process is defined as follows:

(29) $$T=inf\left\{t|x_{N,M}\geq \omega \right\}$$

For the Wiener degradation process model described in Equation (4) of Section 2.2, the distribution of ω is given using the IG distribution. From the reliability definition $R\left(t\right)=P\left(T>t\right)$, its expression is as shown in Equation (30):

(30) $$R^{Wiener}\left(t\right)=P\left(T>t\right)=1-\Phi \left(\frac{\eta t-\omega }{\sigma \sqrt{t}}\right)-exp\left(\frac{2\eta \omega }{\sigma^{2}}\right)\Phi \left(-\frac{\eta t+\omega }{\sigma \sqrt{t}}\right)$$

According to $F\left(t\right)=P\left(T\leq t\right)=1-R\left(t\right)$, the explicit expression of the CDF can be obtained, as shown in Equation (31). By differentiating $F\left(t\right)$ with respect to time t, the PDF $f\left(t\right)$ can be obtained, and its mathematical expression is shown in Equation (32):

(31) $$F^{Wiener}\left(t\right)=P\left(T\leq t\right)=1-R^{Wiener}\left(t\right)=\Phi \left(\frac{\eta t-\omega }{\sigma \sqrt{t}}\right)+exp\left(\frac{2\eta \omega }{\sigma^{2}}\right)\Phi \left(-\frac{\eta t+\omega }{\sigma \sqrt{t}}\right)$$

(32) $$f^{Wiener}\left(t\right)=\frac{{dF}^{Wiener}\left(t\right)}{dt}=\frac{\omega }{\sqrt{2\pi \sigma^{2}t^{3}}}exp\left(-\frac{{\left(\omega -\eta t\right)}^{2}}{2\sigma^{2}t}\right)$$

### 4.2. IG Process Model

For the IG degradation process model described in Equation (5) of Section 2.2, its FPT follows the IG distribution as well, which is suitable for characterizing the probabilistic relationship between FPT and the failure threshold ω. Similarly, according to the reliability definition, the reliability function under the IG degradation model can be obtained, as shown in Equation (33):

(33) $$R^{IG}\left(t\right)=P\left(T>t\right)=1-\Phi \left(\sqrt{\frac{\lambda \omega }{t}}\left(\frac{\mu t}{\omega }-1\right)\right)-exp\left(\frac{2\lambda }{\mu }\right)\Phi \left(-\sqrt{\frac{\lambda \omega }{t}}\left(\frac{\mu t}{\omega }+1\right)\right)$$

Similar to the steps in the Wiener degradation process model, the CDF and PDF expressions for the IG degradation process model can be obtained from the reliability function expression, as shown in Equations (34) and (35):

(34) $$F^{IG}\left(t\right)=P\left(T\leq t\right)=1-R^{IG}\left(t\right)\;\;=\Phi \left(\sqrt{\frac{\lambda \omega }{t}}\left(\frac{\mu t}{\omega }-1\right)\right)+exp\left(\frac{2\lambda }{\mu }\right)\Phi \left(-\sqrt{\frac{\lambda \omega }{t}}\left(\frac{\mu t}{\omega }+1\right)\right)$$

(35) $$f^{IG}\left(t\right)=\frac{{dF}^{IG}\left(t\right)}{dt}=\sqrt{\frac{\lambda \omega }{2\pi t^{3}}}exp\left(-\frac{{\lambda \left(\omega -\mu t\right)}^{2}}{2\mu^{2}\omega t}\right)$$

### 4.3. Two-Phase Hybrid Stochastic Degradation Process Model

Because the degradation process consists of two distinct phases, the reliability function must be calculated separately for each phase. Subsequently, overall system reliability for the two consecutive phases is derived. The corresponding derivations and expressions of the CDF and PDF for both phases are presented. According to the modeling assumptions described above, the first phase degradation process follows a normal distribution, whereas the second phase degradation process follows the IG distribution. Consequently, the system reliability analysis is conducted by partitioning the time domain into two intervals based on the change point moment q.

For the first phase, $0\;{\leq t\leq t}_{q}$, the FPT from the start to the threshold ω is denoted as $T_{1}$. As the degradation behavior in the first phase is entirely determined by the Wiener process, Equation (30) is the system reliability expression for the first phase, i.e.,

(36) $${R^{1}\left(t\right)=P\left(T_{1}>t\right)=R}^{Wiener}\left(t\right)\;=1-\Phi \left(\frac{\eta^{*}t-\omega }{\sigma^{*}\sqrt{t}}\right)-exp\left(\frac{2\eta^{*}\omega }{{\left(\sigma^{2}\right)}^{*}}\right)\Phi \left(-\frac{\eta^{*}t+\omega }{\sigma^{*}\sqrt{t}}\right),\;0\;{\leq t\leq t}_{q}$$

After obtaining the reliability function $R^{1}\left(t\right)$ for the first phase, its corresponding CDF and PDF are provided. The system expressions for the CDF and PDF of the first phase of the model are also equivalent to the CDF and PDF of the Wiener process, i.e.,

(37) $${F^{1}\left(t\right)=P\left(T_{1}\leq t\right)=F}^{Wiener}\left(t\right)=\Phi \left(\frac{\eta^{*}t-\omega }{\sigma^{*}\sqrt{t}}\right)+exp\left(\frac{2\eta^{*}\omega }{{\left(\sigma^{2}\right)}^{*}}\right)\Phi \left(-\frac{\eta^{*}t+\omega }{\sigma^{*}\sqrt{t}}\right)$$

(38) $${f^{1}\left(t\right)=f}^{Wiener}\left(t\right)=\frac{\omega }{\sqrt{2\pi {\left(\sigma^{2}\right)}^{*}t^{3}}}exp\left(-\frac{{\left(\omega -\eta^{*}t\right)}^{2}}{2{\left(\sigma^{2}\right)}^{*}t}\right)$$

The system degradation process transitions from the Wiener process to the IG process as soon as the time exceeds the change point $t_{q}$. The second phase’s reliability depends on both the survival probability of the first phase and the conditional probability distribution of the degradation amount $X_{q}$ given survival up to the change point. The reliability of the second phase needs to be synthesized based on the conditional reliability under different initial degradation conditions.

If the product does not fail during the first phase, the degradation level at the change point, denoted by $X_{q}$, follows the conditional probability density function $f_{X\left(t_{q}\right)}\left(x_{q}|x_{q}<\omega \right)$. Conditional distribution characterizes the initial condition of the system upon entering the second degradation phase, as expressed in Equation (39).

(39) $$f_{X\left(t_{q}\right)}\left(x_{q}|x_{q}<\omega \right)=\frac{1}{\sqrt{2\pi {\left(\sigma^{2}\right)}^{*}t_{q}}}\;\;\cdot \frac{exp\left(-\frac{{\left(x_{q}-\eta^{*}t_{q}\right)}^{2}}{2{\left(\sigma^{2}\right)}^{*}t_{q}}\right)-exp\left(\frac{2\eta^{*}\omega }{{\left(\sigma^{2}\right)}^{*}}\right)exp\left(-\frac{{\left(x_{q}+\eta^{*}t_{q}-2\omega \right)}^{2}}{2{\left(\sigma^{2}\right)}^{*}t_{q}}\right)}{R^{Wiener}\left(t_{q}\right)}$$

The second phase is the IG process. $T_{2}$ denotes the FPT from $t_{q}$ until the degradation trajectory reaches the threshold ω and $X\left(t_{q}\right)=x_{q}$ with $x_{q}<\omega$ representing the degradation amount at time $t_{q}$. The conditional reliability $R^{IG}\left({t-t}_{q}|\omega -x_{q}\right)$ is expressed as Equation (40):

(40) $$R^{IG}\left({t-t}_{q}|\omega -x_{q}\right)=P\left(T_{2}>t|X\left(t_{q}\right)<\omega \right)\;\;=1-\Phi \left(\sqrt{\frac{\lambda^{*}}{t-t_{q}}}\left(\frac{t-t_{q}}{\mu^{*}}\right)-\left(\omega -x_{q}\right)\right)\;\;-exp\left(\frac{2\lambda^{*}\left(\omega -x_{q}\right)}{\mu^{*}}\right)\cdot \Phi \left(-\sqrt{\frac{\lambda^{*}}{t-t_{q}}}\left(\frac{t-t_{q}}{\mu^{*}}+\left(\omega -x_{q}\right)\right)\right)$$

The overall reliability of the second phase is $R^{2}\left(t\right)=P\left(T>t\right)$. The product must survive until time $t_{q}$ in the first phase and until time t in the second phase. Thus, the overall reliability function of the second phase is provided by the law of total probability, as shown in Equation (41):

(41) $$R^{2}\left(t\right)=P\left(T>t|X\left(t_{q}\right)<\omega \right)=P\left(X\left(t_{q}\right)<\omega \right)\cdot P\left(T_{2}>t|X\left(t_{q}\right)<\omega \right)$$

where $P\left(X\left(t_{q}\right)<\omega \right)=R^{Wiener}\left(t_{q}\right)$. Through Equation (39), the overall reliability expression for the second phase can be obtained, as shown in Equation (42):

(42) $$R^{2}\left(t\right)=P\left(T>t|X\left(t_{q}\right)<\omega \right)\;\;=R^{Wiener}\left(t_{q}\right)\cdot \int_{0}^{\omega }\begin{array}{c} f_{X\left(t_{q}\right)}\left(x_{q}|x_{q}<\omega \right)\\ \cdot P\left(T_{2}>t|X\left(t_{q}\right)=x_{q},x_{q}<\omega \right)dx_{q} \end{array}\;\;=R^{Wiener}\left(t_{q}\right)\cdot \int_{0}^{\omega }{f_{X\left(t_{q}\right)}\left(x_{q}|x_{q}<\omega \right)\cdot R}^{IG}\left({t-t}_{q}|\omega -x_{q}\right)dx_{q},t_{q}<t$$

Substituting Equations (39) and (40) into Equation (42), the complete expression for $R^{2}\left(t\right)$ is shown in Equation (43):

(43) $${R^{2}\left(t\right)=R}^{Wiener}\left(t_{q}\right)\;\;\cdot \int_{0}^{\omega }\frac{1}{\sqrt{2\pi {\left(\sigma^{2}\right)}^{*}t_{q}}}\cdot \frac{exp\left(-\frac{{\left(x_{q}-\eta^{*}t_{q}\right)}^{2}}{2{\left(\sigma^{2}\right)}^{*}t_{q}}\right)-exp\left(\frac{2\eta^{*}\omega }{{\left(\sigma^{2}\right)}^{*}}\right)exp\left(-\frac{{\left(x_{q}+\eta^{*}t_{q}-2\omega \right)}^{2}}{2{\left(\sigma^{2}\right)}^{*}t_{q}}\right)}{R^{Wiener}\left(t_{q}\right)}\;\;\cdot R^{IG}\left({t-t}_{q}|\omega -x_{q}\right)dx_{q}\;\;=\int_{0}^{\omega }\frac{1}{\sqrt{2\pi {\left(\sigma^{2}\right)}^{*}t_{q}}}\cdot \left[exp\left(-\frac{{\left(x_{q}-\eta^{*}t_{q}\right)}^{2}}{2{\left(\sigma^{2}\right)}^{*}t_{q}}\right)-exp\left(\frac{2\eta^{*}\omega }{{\left(\sigma^{2}\right)}^{*}}\right)exp\left(-\frac{{\left(x_{q}+\eta^{*}t_{q}-2\omega \right)}^{2}}{2{\left(\sigma^{2}\right)}^{*}t_{q}}\right)\right]\;\;\cdot \left[\begin{array}{c} 1-\Phi \left(\sqrt{\frac{\lambda^{*}}{t-t_{q}}}\left(\frac{t-t_{q}}{\mu^{*}}\right)-\left(\omega -x_{q}\right)\right)-\exp\left(\frac{2\lambda^{*}\left(\omega -x_{q}\right)}{\mu^{*}}\right)\cdot \\ \Phi \left(-\sqrt{\frac{\lambda^{*}}{t-t_{q}}}\left(\frac{t-t_{q}}{\mu^{*}}+\left(\omega -x_{q}\right)\right)\right) \end{array}\right]dx_{q}$$

Based on the first phase’s reliability function, obtained through Equations (36) and (43), the overall reliability function expression of the two-phase hybrid stochastic degradation process model is shown in Equation (44):

(44) $$R_{\mathrm{Two}\text{-}\mathrm{phase}}\left(t\right)=\;\;\left\{\begin{array}{c} 1-\Phi \left(\frac{\eta^{*}t-\omega }{\sigma^{*}\sqrt{t}}\right)-\exp\left(\frac{2\eta^{*}\omega }{{\left(\sigma^{2}\right)}^{*}}\right)\Phi \left(-\frac{\eta^{*}t+\omega }{\sigma^{*}\sqrt{t}}\right),\;\;\;0\;{\leq t\leq t}_{q}\\ \int_{0}^{\omega }\frac{1}{\sqrt{2\pi {\left(\sigma^{2}\right)}^{*}t_{q}}}\cdot \left[exp\left(-\frac{{\left(x_{q}-\eta^{*}t_{q}\right)}^{2}}{2{\left(\sigma^{2}\right)}^{*}t_{q}}\right)-exp\left(\frac{2\eta^{*}\omega }{{\left(\sigma^{2}\right)}^{*}}\right)exp\left(-\frac{{\left(x_{q}+\eta^{*}t_{q}-2\omega \right)}^{2}}{2{\left(\sigma^{2}\right)}^{*}t_{q}}\right)\right]\\ \cdot \left[\begin{array}{c} 1-\Phi \left(\sqrt{\frac{\lambda^{*}}{t-t_{q}}}\left(\frac{t-t_{q}}{\mu^{*}}\right)-\left(\omega -x_{q}\right)\right)-\exp\left(\frac{2\lambda^{*}\left(\omega -x_{q}\right)}{\mu^{*}}\right)\\ \Phi \left(-\sqrt{\frac{\lambda^{*}}{t-t_{q}}}\left(\frac{t-t_{q}}{\mu^{*}}+\left(\omega -x_{q}\right)\right)\right) \end{array}\right]dx_{q},\;\;\;t_{q}<t \end{array}\right.$$

According to Equation (44), the overall CDF and PDF expressions of the two-phase hybrid stochastic degradation process model are shown in Equations (45) and (46), respectively:

(45) $$F_{\mathrm{Two}\text{-}\mathrm{phase}}\left(t\right)=1-R_{\mathrm{Two}\text{-}\mathrm{phase}}\left(t\right)=\;\;\left\{\begin{array}{c} \Phi \left(\frac{\eta^{*}t-\omega }{\sigma^{*}\sqrt{t}}\right)+\exp\left(\frac{2\eta^{*}\omega }{{\left(\sigma^{2}\right)}^{*}}\right)\Phi \left(-\frac{\eta^{*}t+\omega }{\sigma^{*}\sqrt{t}}\right),\;\;\;0\;{\leq t\leq t}_{q}\\ \Phi \left(\frac{\eta^{*}t_{q}-\omega }{\sigma^{*}\sqrt{t_{q}}}\right)+\exp\left(\frac{2\eta^{*}\omega }{{\left(\sigma^{2}\right)}^{*}}\right)\Phi \left(-\frac{\eta^{*}t_{q}+\omega }{\sigma^{*}\sqrt{t_{q}}}\right)+\\ \int_{0}^{\omega }\frac{1}{\sqrt{2\pi {\left(\sigma^{2}\right)}^{*}t_{q}}}\cdot \left[exp\left(-\frac{{\left(x_{q}-\eta^{*}t_{q}\right)}^{2}}{2{\left(\sigma^{2}\right)}^{*}t_{q}}\right)-exp\left(\frac{2\eta^{*}\omega }{{\left(\sigma^{2}\right)}^{*}}\right)exp\left(-\frac{{\left(x_{q}+\eta^{*}t_{q}-2\omega \right)}^{2}}{2{\left(\sigma^{2}\right)}^{*}t_{q}}\right)\right]\\ \cdot \left[\begin{array}{c} \Phi \left(\sqrt{\frac{\lambda^{*}}{t-t_{q}}}\left(\frac{t-t_{q}}{\mu^{*}}\right)-\left(\omega -x_{q}\right)\right)+\exp\left(\frac{2\lambda^{*}\left(\omega -x_{q}\right)}{\mu^{*}}\right)\\ \Phi \left(-\sqrt{\frac{\lambda^{*}}{t-t_{q}}}\left(\frac{t-t_{q}}{\mu^{*}}+\left(\omega -x_{q}\right)\right)\right) \end{array}\right]dx_{q},\;\;\;t_{q}<t \end{array}\right.$$

(46) $$f_{\mathrm{Two}\text{-}\mathrm{phase}}\left(t\right)=\frac{{dF}_{\mathrm{Two}\text{-}\mathrm{phase}}\left(t\right)}{dt}=\;\;\left\{\begin{array}{c} \frac{\omega }{\sqrt{2\pi {\left(\sigma^{2}\right)}^{*}t^{3}}}\exp\left(-\frac{{\left(\omega -\eta^{*}t\right)}^{2}}{2{\left(\sigma^{2}\right)}^{*}t}\right),\;\;\;0\;{\leq t\leq t}_{q}\\ \int_{0}^{\omega }\frac{1}{\sqrt{2\pi {\left(\sigma^{2}\right)}^{*}t_{q}}}\cdot \left[exp\left(-\frac{{\left(x_{q}-\eta^{*}t_{q}\right)}^{2}}{2{\left(\sigma^{2}\right)}^{*}t_{q}}\right)-exp\left(\frac{2\eta^{*}\omega }{{\left(\sigma^{2}\right)}^{*}}\right)exp\left(-\frac{{\left(x_{q}+\eta^{*}t_{q}-2\omega \right)}^{2}}{2{\left(\sigma^{2}\right)}^{*}t_{q}}\right)\right]\\ \cdot \sqrt{\frac{\lambda^{*}}{2\pi {\left(t-t_{q}\right)}^{3}}}\cdot \exp\left(-\frac{\lambda^{*}{\left[\left(\omega -x_{q}\right)-\mu^{*}\left(t-t_{q}\right)\right]}^{2}}{2{\left(\mu^{*}\right)}^{2}\left(t-t_{q}\right)}\right)dx_{q},\;\;\;t_{q}<t \end{array}\right.$$

## 5. A Numerical Example

**Figure 1:**
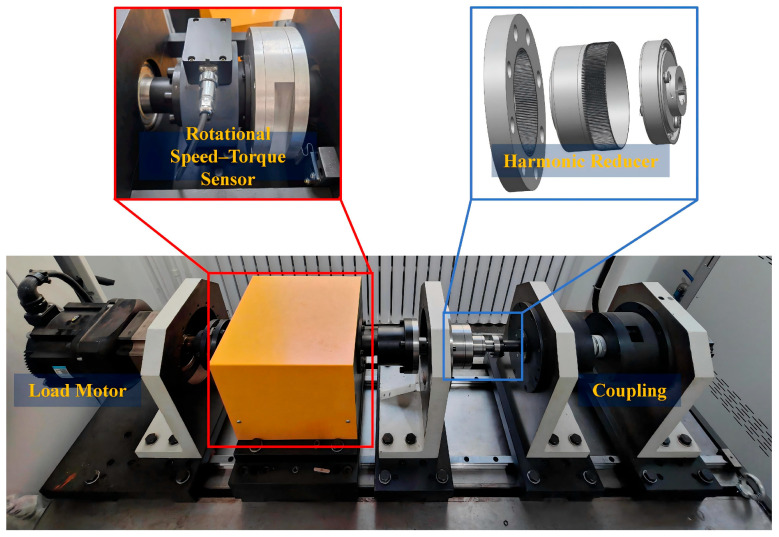
Test platform for harmonic reducers.

**Figure 2:**
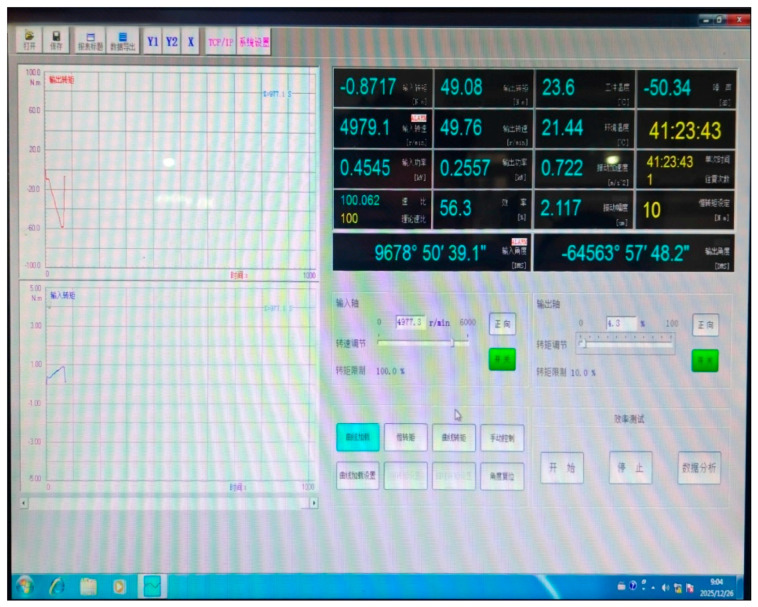
Monitoring system for harmonic reducers.

The following numerical example is based on degradation test data collected from five harmonic reducers produced by a certain enterprise. According to the technical specifications, transmission efficiency during operation is selected as the performance degradation indicator to reflect the overall transmission performance loss caused by cumulative wear and damage. The failure threshold is defined as the point at which the transmission efficiency decreases to 50% [37,41]. The degradation test was conducted using a reliability test device consisting of a test platform and a monitoring system, as shown in Figure 1 and Figure 2.

**Figure 3:**
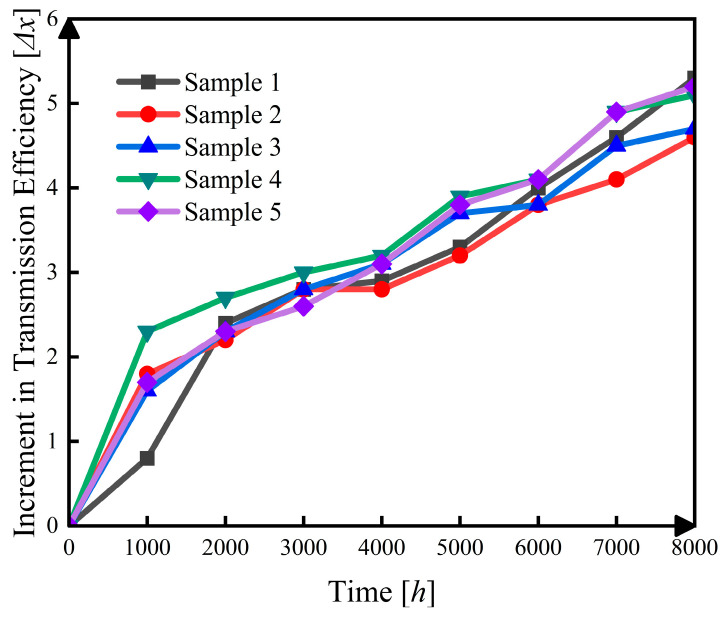
Incremental changes in transmission efficiency across harmonic reducers.

During the test, the integrated speed–torque sensor measured the input and output speed and torque, and the corresponding transmission efficiency was calculated according to Equation (47). No sudden failure occurred in any sample during observation, and the degradation trajectories of all samples remained above the prescribed failure threshold. In total, five harmonic reducers were tested, and 40 transmission efficiency increment observations were obtained over eight adjacent inspection intervals. These data constitute the basis for change point identification, model fitting, and reliability analysis. Figure 3 presents the transmission efficiency increment data for the five tested harmonic reducers, where each sequence corresponds to one sample and is constructed from observations over adjacent inspection intervals.

(47) $$x=\frac{A_{Output}}{A_{Input}}\times 100\%=\frac{B_{Output}\cdot C_{Output}}{B_{Input}\cdot C_{Input}}\times 100\%$$

where A,B, and C denote power, torque, and rotational speed, respectively.

To verify the rationality of the independent increment assumption in the Wiener and IG process models used in this paper, a statistical test of the potential serial correlation of performance degradation increment sequences was performed. Detailed calculation steps for the auto-correlation test are provided in Appendix A. The results show that within the examined lag order range, the auto-correlation coefficients of all tested products failed the significance test (p>0.05), and no statistically significant serial correlation was found between the performance degradation increments. Therefore, under the test conditions and sample size considered in this paper, the independent increment assumption of the Wiener and IG processes is not negated by the increment data, and it can serve as a reasonable premise for subsequent parameter estimation and reliability analysis.

### 5.1. Judgment and Confirmation of the Change Point

According to the SIC-based change point identification and decision rule proposed in Section 2.3, change points are first estimated for each sample individually. The consistency of the estimated results across samples is then examined to determine whether a unified change point or sample-level change points should be adopted in the subsequent analysis.

The $\Delta t_{N,M}=1000$ h values were used for the Wiener and IG process models. Parameter estimation, log-likelihood function, and corresponding SIC values for the two models are shown in Table 1 and Table 2. The SIC value of the Wiener process model for each sample is smaller than the SIC value of the IG process model for that sample. Therefore, $H_{0}^{Wiener}$ was chosen as the hypothesis for $H_{0}$.

In the two-phase hybrid stochastic degradation process model, the test interval was fixed at $\Delta \tau_{1}\left(t_{N,M}\right)=\Delta \tau_{2}\left(t_{N,M}\right)=1000h$. Table 3 provides the parameter estimation results, while Table 4 presents the log-likelihood values together with the corresponding SIC values. The change point was identified by searching through a discrete set of candidate locations corresponding to the inspection intervals. Therefore, the estimated change point locations are integer candidate values. As shown in Table 4, the SIC values for all five harmonic reducers reach their minimum at the same candidate location, ${\hat{q}}_{N}=6$, indicating that the two-phase model with $t_{N,M}=6000h$ provides the best fit for the data. This consistency in SIC-based model selection across samples indicates that the unified change point is supported by the data, rather than being imposed a priori. This time point marks the transition of the degradation process from a stable wear phase to an accelerated degradation phase. Consequently, the alternative hypothesis $H_{1,t_{q}\;=\;6000}$ was adopted.

**Figure 4:**
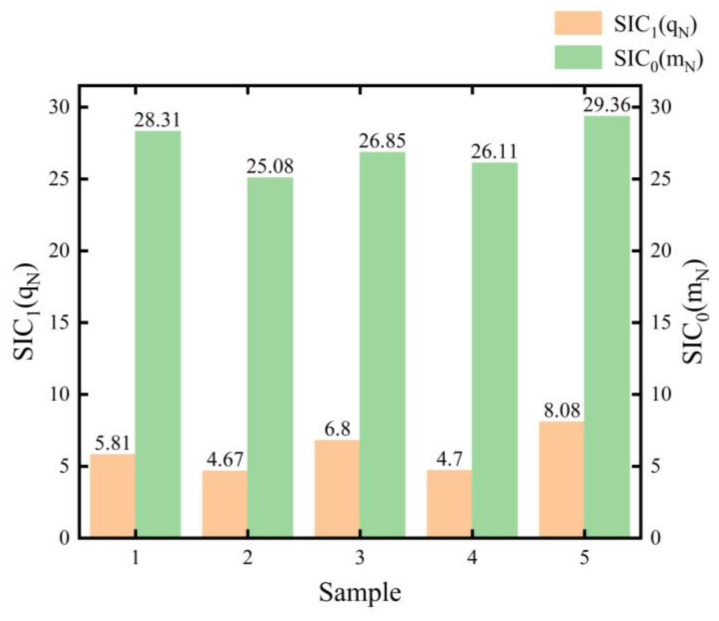
Comparison of SIC values for 5 samples under the proposed model and the traditional model.

As shown in Table 5 and Table 6, the $\frac{\eta }{\sigma^{2}}$ values estimated for all samples are greater than 1, demonstrating that it is reasonable to ignore negative increments. In addition, as shown in Figure 4, the change points of ${SIC}_{Two\text{-}phaseN}^{*}\left({\hat{q}}_{N}\right)$ and ${SIC}_{N}^{Wiener}\left(m_{N}\right)$ for the 5 samples are represented by ${SIC}_{1}\left(q_{N}\right)$ and ${SIC}_{0}\left(m_{N}\right)$, respectively. For each sample, the SIC value obtained from the traditional models is larger than the minimum SIC value corresponding to the proposed two-phase hybrid stochastic degradation process model. Specifically, $H_{0}^{Wiener}>minH_{1,t_{q}=6000}$, indicating that a change point exists at t=6000 h for each sample. Consequently, the null hypothesis $H_{0}$ was rejected, and the alternative hypothesis $H_{1,t_{q}}$ was accepted.

Table 7 compares the computation time required for parameter estimation of different degradation models using the same hardware. The computation process includes both parameter estimation and evaluation of the corresponding log-likelihood functions. As additional sample-level change point identification is needed, the proposed two-phase model requires greater computation time than the Wiener and IG process models. However, because parameter estimation for each phase is based on existing closed-form solutions and the change point search is one-dimensional, the proposed model does not introduce significantly greater computation time.

### 5.2. Sensitivity Analysis of SIC to Small Samples

Although SIC performs well in change point detection, its sensitivity to small samples requires further investigation. The expression for SIC is shown in Equation (1). From the perspective of sensitivity analysis, the sensitivity of SIC to small samples is mainly due to its penalty term Vlnm. Therefore, the sensitivity coefficient $\frac{V}{m}$ of Vlnm relative to the sample size m is obtained through Equation (48):

(48) $$\frac{\partial V\ln m}{\partial m}=\frac{V}{m}$$

It can be seen that when the sample size is small in the initial stage of testing, $\frac{V}{m}$ has a large value, indicating that SIC is sensitive to changes in m. When the sample size m is small, change point identification may be conservative. Although the value of the log-likelihood function lnL changes with the sample size as well, its contribution to SIC usually increases linearly with the increase in data volume. Conversely, as the sample size increases, the influence of the penalty term weakens, and lnL gradually becomes dominant. Therefore, the sensitivity of the penalty term Vlnm decreases rapidly with the increase in m, which helps clarify the behavior of SIC under small-sample conditions and provides a clearer basis for interpreting the identified change point.

### 5.3. Hypothesis Testing and Goodness of Fit Testing

Assuming the degradation process follows Wiener and IG processes, the overall parameters of the model can be estimated as ${\hat{\Theta }}^{Wiener}=\left(\hat{\eta },{\hat{\sigma }}^{2}\right)=\left(3.4\times 10^{-3},1.08\times 10^{-3}\right)$ and ${\hat{\Theta }}^{IG}=\left(\mu ,\lambda \right)=\left(4.98\times 10^{-3},1.55\times 10^{-3}\right)$. For the two-phase hybrid stochastic degradation process model, the overall parameters can be estimated as ${\hat{\Theta }}_{Two\text{-}phase}^{*}=\left({\hat{\eta }}^{*},{\left({\hat{\sigma }}^{2}\right)}^{*},{\hat{\mu }}^{*},{\hat{\lambda }}^{*}\right)=\left(2.93\times 10^{-3},5.37\times 10^{-4},4.97\times 10^{-3},1.54\times 10^{-3}\right)$. According to the change point at $t_{q}=6000\;h$, the first 6 intervals are classified as Wiener processes, assuming that the increments in this phase follow a normal distribution. The last 2 intervals are classified as IG processes, assuming that the increments in this phase follow an IG distribution. To conduct a feasible analysis, it is necessary to test whether the three process models conform to the observed degradation data. This paper uses the Kolmogorov–Smirnov (K-S) test to evaluate whether the models conform to this distribution. The null hypothesis is set as $H_{original}$*: the sample values follow the assumed theoretical distribution*, with an asymptotic significance level of $p_{original}=0.05$. The test results are shown in Table 8, Table 9 and Table 10, respectively. The Wiener process model and the two-phase model cannot reject the null hypothesis $H_{original}$, indicating that both models are statistically consistent with the observed data under the K-S criterion. In the second phase of the two-phase hybrid model, the result is considered supportive and interpreted alongside the comparative model’s performance, the observed accelerated degradation trend after the change point, and the reliability assessment results. However, the IG process model failed the K-S test. It cannot effectively characterize the degradation law of the harmonic reducer’s transmission efficiency and cannot be used as a degradation model.

**Figure 5:**
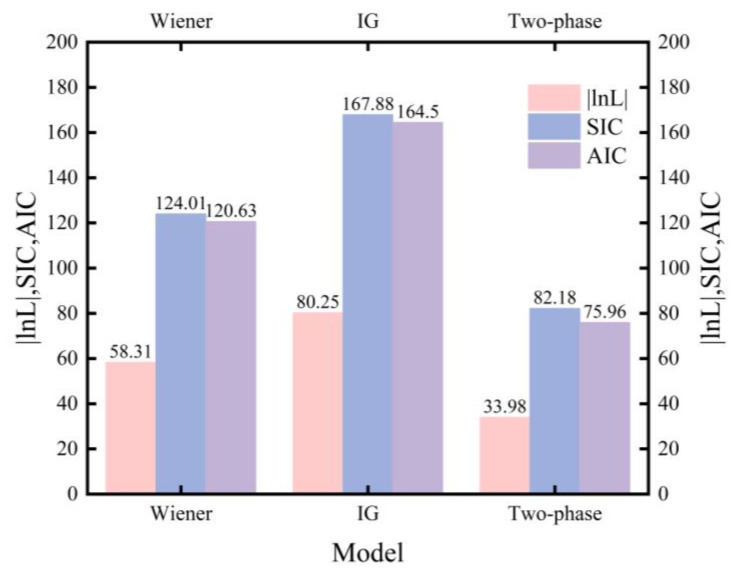
The $\left|\ln L\right|$, SIC, and AIC values of the Wiener, IG, and two-phase hybrid stochastic process models.

Although the IG process model rejects the null hypothesis, it can still be used as a control model for goodness of fit, as subsequent model evaluation focuses on its ability to fit the observed data rather than satisfying the actual distribution form. In this paper, SIC was used for model selection and to determine whether a change point exists. It can also be used here to evaluate the goodness of fit of multiple candidate models. Furthermore, the Akaike Information Criterion (AIC) was used to evaluate the goodness of the model [42] using the formula AIC=2V−2lnL. Figure 5 shows the $\left|\ln L\right|$, SIC, and AIC values of each model. Compared to the Wiener process model and the IG model, the proposed model has the smallest $\left|\ln L\right|$, SIC, and AIC values, and each value is significantly different from the other two models. The proposed model not only provides stronger statistical interpretability for degradation data but also achieves a more appropriate trade-off between goodness of fit and model complexity. Consequently, the two-phase hybrid stochastic degradation process model proposed in this paper is well suited for characterizing the phase-dependent performance degradation behavior of harmonic reducers.

### 5.4. Reliability Assessment and Lifetime Analysis

**Figure 6:**
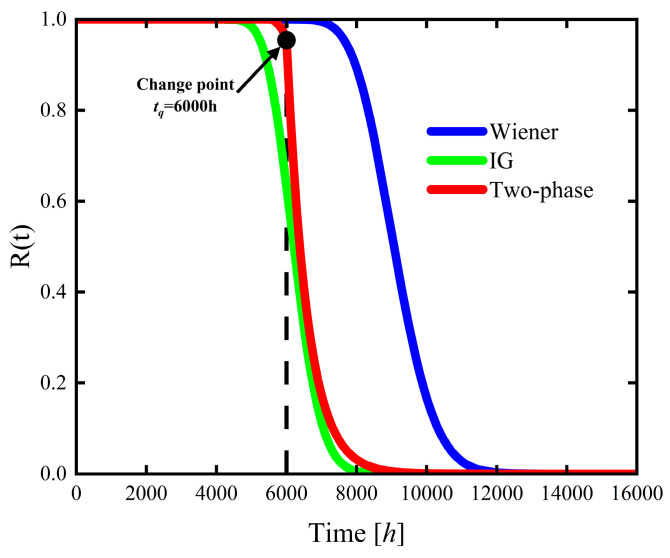
Estimated reliability functions of the product’s lifetime based on the Wiener, IG, and two-phase hybrid process models.

**Figure 7:**
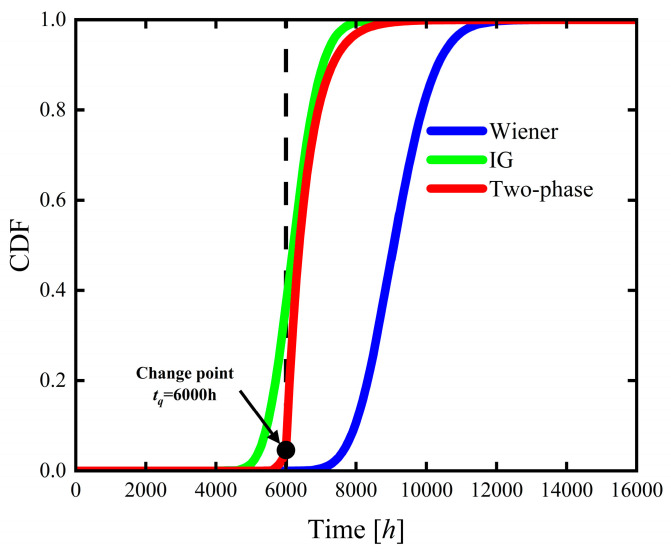
Estimated CDFs of the product’s lifetime based on the Wiener, IG, and two-phase hybrid process models.

**Figure 8:**
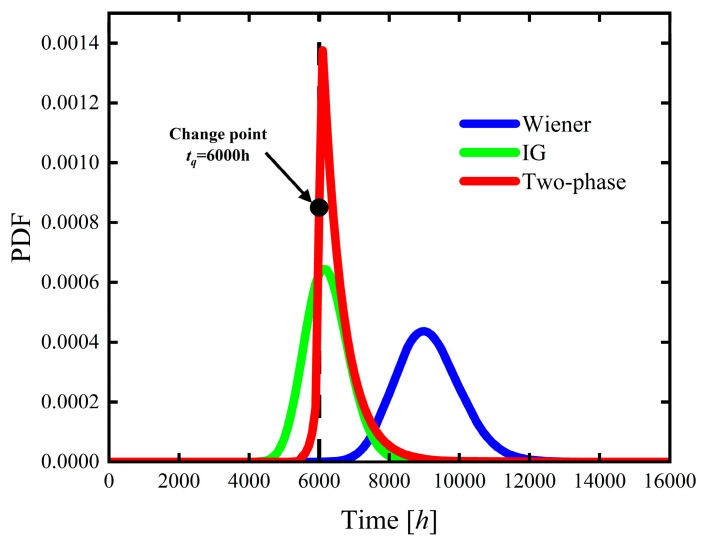
Estimated PDFs of the product’s lifetime based on the Wiener, IG, and two-phase hybrid process models.

This section compares the Wiener process model, the IG process model, and the two-phase hybrid stochastic degradation process model in terms of reliability assessment and lifetime distribution characteristics by analyzing their reliability functions, CDFs, and PDFs. The corresponding reliability functions, CDFs, and PDFs of the three models are presented in Figure 6, Figure 7 and Figure 8.

As shown in Figure 6, compared with the two-phase hybrid stochastic degradation process model, the reliability curves of the Wiener and IG process models exhibit a more gradual downward trend. Before the change point at $t_{q}=6000\;h$, the IG process model shows the fastest decline in reliability, whereas the reliability variations in the Wiener model and the two-phase hybrid model are negligible. After the change point, the reliability of the two-phase hybrid stochastic degradation process model decreases significantly faster than that of the other two models, indicating that this model effectively captures the accelerated degradation phase of the product, leading to the shorter post-change point lifetime. In addition, the reliability trend of the IG process model is closer to that of the two-phase hybrid model, with the reliability of both models approaching 0 at approximately t=8000 h. However, the IG process model neglects the transitional phase from cumulative performance degradation to accelerated degradation, rendering it inadequate for accurately characterizing the actual reliability behavior of harmonic reducers. In contrast, the reliability of the Wiener process model remains greater than 0.8 at this time point, overestimating the actual reliability of the product.

The differences in degradation trends among the tested products are further illustrated by the CDF and PDF. As shown in Figure 7, the CDF of the two-phase hybrid stochastic degradation process model increases rapidly after the change point at $t_{q}=6000\;h$, reaching a high failure probability at an earlier time. The CDF trend of the IG process model is relatively similar in the post-change point phase, and the CDFs of both models approach 1 at approximately t=8000 h. In contrast, the Wiener process model yields a CDF value of less than 0.2 at this time point, indicating a pronounced delay in failure time prediction. The result demonstrates that the Wiener model fails to capture the lifetime concentration effect induced by the accelerated degradation behavior after the change point.

As shown in Figure 8, the PDF of the two-phase hybrid stochastic degradation process model exhibits a pronounced peak in the vicinity of the change point at $t_{q}=6000\;h$, indicating a rapid increase in product failure risk. This indicates that the proposed model can reflect the transition from gradual degradation to accelerated degradation more reasonably than the single-process models during actual service. Degradation before the change point corresponds to a relatively stable wear stage in which lubrication and elastic buffering effects are still effective, and transmission efficiency decreases slowly with relatively strong stochastic fluctuation characteristics. After the change point, however, cumulative wear becomes more pronounced, the contact condition gradually deteriorates, and degradation evolves into a more monotonic and accelerated process. Therefore, the two-phase model is physically consistent with the actual degradation evolution of harmonic reducers. Although the PDF trend of the IG process model is more comparable after the change point, its peak value is substantially lower than that of the two-phase hybrid model, and its distribution is considerably more dispersed. In comparison, the Wiener process model exhibits a more pronounced rightward shift in the peak PDF location together with a broader distribution, which reflects a slow variation in failure risk, leading to underestimation of early failure probability and corresponding overestimation of product lifetime.

The time to actual failure corresponding to a reliability level of $R\left(t\right)=0.01$ for the three process models is summarized in Table 11. To verify the accuracy of the results, the time to actual failure of the same type of harmonic reducer monitored in the laboratory for a period of 8690 h was compared to the reliability evaluation results. The evaluation deviation is defined as $Error=\frac{\left|t_{Pred}\;-\;t_{Obs}\right|}{t_{Obs}}\times 100\%$, where $t_{Pred}$ is the predicted lifetime obtained through process models and $t_{Obs}$ is the observed lifetime. We obtained assessment biases of 32%, 9.9%, and 0.06% for the three models. Among the three candidate models, the two-phase hybrid stochastic degradation process model yields the smallest reliability assessment bias and shows the closest agreement with the observed failure time. These results suggest that the proposed model provides a more reasonable basis for reliability assessment and lifetime distribution analysis under the present data conditions.

## 6. Conclusions

To address the complex performance degradation characteristics of harmonic reducers during long-term service, this paper proposes a two-phase hybrid stochastic degradation framework and develops a corresponding change point stochastic degradation model. Based on actual degradation data, maximum likelihood estimation was employed to estimate the model’s parameters. In the proposed model, the Wiener process was used to characterize slow early-phase degradation behavior, while the IG process was adopted to describe accelerated later-phase degradation behavior. In comparison with traditional Wiener and IG process models, the existence and location of the change point were identified. Hypothesis testing was conducted for the three models, and their lnL, AIC, and SIC values were systematically compared. The results indicate that the proposed model provides a better overall fit than the traditional Wiener and IG models under the current dataset. These findings further substantiate that the two-phase hybrid stochastic degradation process model, which includes a change point structure, can more reasonably represent the phase-dependent performance degradation attributes of harmonic reducers.

Based on this framework, analytical expressions for the reliability function, CDF, and PDF of the proposed model were derived for the phases before and after the change point, and the model was subsequently validated. Compared with the Wiener and the IG process models, the proposed model consistently exhibits pronounced rapid failure characteristics after the change point across all three functions. In contrast, the Wiener process model systematically overestimates the product lifetime, while the IG process model fails to capture the transition mechanism from early accumulated damage to accelerated degradation. Consequently, both single-process models show limitations in characterizing the degradation behavior of harmonic reducers compared to the proposed two-phase model.

Overall, the two-phase hybrid stochastic degradation process model proposed in this paper provides a statistical modeling framework that is more consistent with actual operating conditions and offers improved physical interpretability for harmonic reducer reliability assessment. The proposed model was developed to assess the degradation behavior of system-level harmonic reducers and is not restricted to the specific product tested in this study. For harmonic reducers of different sizes or brands, the framework remains applicable provided that the corresponding degradation data are used to recalculate and validate the model’s parameters for the target product. In addition, the parameter estimates in this study were obtained from degradation data for only five harmonic reducers. Under such small-sample conditions, the estimates may exhibit relatively high variance, and the corresponding quantitative results should therefore be interpreted with caution. In future work, uncertainty quantification for the reliability function, such as profile-likelihood-based or bootstrap-based confidence intervals, will be considered to more explicitly characterize the statistical uncertainty associated with the predictions. The proposed framework also establishes a solid foundation for future research on remaining useful life prediction, condition assessment, and optimal test design, while further validation using larger datasets and multiple operating conditions will be an important direction for future work.

## Figures and Tables

**Table 1:** Estimation results of the Wiener process model.

Sample	${\hat{\eta }}_{N}$	${\hat{\sigma }}_{N}^{2}$	${\ln\hat{L}}_{N}^{Wiener}$	${SIC}_{N}^{Wiener}\left(m_{N}\right)$
1	0.00338	0.00119	−12.08	28.31
2	0.00316	0.0008	−10.46	25.08
3	0.00331	0.00099	−11.35	26.85
4	0.0036	0.00091	−10.97	26.11
5	0.00346	0.00136	−12.6	29.36

**Table 2:** Estimation results of the IG process model.

Sample	${\hat{\mu }}_{N}$	${\hat{\lambda }}_{N}$	${\ln\hat{L}}_{BasicN}^{IG}$	${SIC}_{BasicN}^{IG}\left(m_{N}\right)$
1	0.00338	0.00127	−129.55	263.26
2	0.00316	0.00112	−108.87	221.91
3	0.00331	0.00123	−139.87	283.9
4	0.00364	0.0014	−74.68	153.51
5	0.00346	0.00133	−155.47	315.1

**Table 3:** Estimation results of the two-phase hybrid stochastic degradation process model.

Sample	${\hat{q}}_{N}$	${\hat{\eta }}_{N}^{*}$	${\left({\hat{\sigma }}_{N}^{2}\right)}^{*}$	${\hat{\mu }}_{N}^{*}$	${\hat{\lambda }}_{N}^{*}$
1	2	0.0021	0.00009	0.00404	0.00103
	3	0.00233	0.00016	0.00432	0.00113
	4	0.00247	0.00018	0.00466	0.00105
	5	0.00264	0.00025	0.00498	0.0012
	6	0.00286	0.00047	0.0053	0.00126
2	2	0.002	0.00004	0.00372	0.00127
	3	0.00226	0.00016	0.00395	0.00134
	4	0.0024	0.00018	0.00419	0.00119
	5	0.00256	0.00024	0.00437	0.00126
	6	0.00276	0.00041	0.0046	0.00136
3	2	0.00195	0.00012	0.00398	0.00127
	3	0.00223	0.00024	0.0042	0.00138
	4	0.00245	0.00032	0.00436	0.0016
	5	0.0027	0.0005	0.0046	0.001
	6	0.00288	0.00059	0.0047	0.00136
4	2	0.0025	0.00004	0.00426	0.00117
	3	0.00266	0.00008	0.00453	0.00145
	4	0.0028	0.00011	0.00473	0.00129
	5	0.00302	0.00028	0.005	0.00226
	6	0.0032	0.0004	0.0051	0.00245
5	2	0.002	0.00009	0.00424	0.00113
	3	0.0022	0.00014	0.00453	0.00238
	4	0.00242	0.00025	0.00476	0.00336
	5	0.0027	0.0005	0.00508	0.00456
	6	0.00293	0.00069	0.0052	0.00129

**Table 4:** Statistical results of the two-phase hybrid stochastic degradation process model.

Sample	${\hat{q}}_{N}$	$\ln{\hat{L}}_{Two\text{-}phaseN}^{Wiener*}$	$\ln{\hat{L}}_{Two\text{-}phaseN}^{IG*}$	$\ln{\hat{L}}_{Two\text{-}phaseN}^{*}$	${SIC}_{Two\text{-}phaseN}^{*}\left({\hat{q}}_{N}\right)$
1	2	−0.43	−27.68	−28.11	59
	3	−1.59	−12.98	−14.57	33.53
	4	−2.32	−3.57	−5.89	17.34
	5	−3.71	−0.75	−4.46	15.35
	6	−6.26	6.94	0.68	5.81
2	2	0.38	−18.42	−18.04	38.86
	3	−1.59	−6.96	−8.55	21.49
	4	−2.25	−1.05	−3.3	12.13
	5	−3.59	−0.08	−3.67	13.78
	6	−5.9	7.15	1.25	4.67
3	2	−0.74	−11.78	−12.52	27.81
	3	−2.13	−3.59	−5.72	15.83
	4	−3.41	−1.91	−5.32	16.19
	5	−5.4	1.77	−3.63	13.71
	6	−6.94	7.12	0.18	6.8
4	2	0.38	−14.95	−14.57	31.9
	3	−0.51	−4.66	−5.17	14.73
	4	−1.35	−2.24	−3.59	12.72
	5	−3.96	1.77	−2.19	10.83
	6	−5.76	7	1.24	4.7
5	2	−0.43	−18.62	−19.05	40.88
	3	−1.31	−6.14	−7.45	19.28
	4	−2.96	−2.63	−5.59	16.71
	5	−5.4	0.91	−4.49	15.43
	6	−7.42	6.97	−0.45	8.08

**Table 5:** Sample-level estimated $\frac{\eta }{\sigma^{2}}$ values for the Wiener process model.

Sample	$\frac{{\hat{\eta }}_{N}}{{\hat{\sigma }}_{N}^{2}}$
1	2.84
2	2.66
3	3.34
4	3.96
5	2.54

**Table 6:** Sample-level estimated $\frac{\eta }{\sigma^{2}}$ values for the Wiener phase of the two-phase model.

Sample	${\hat{q}}_{N}$	$\frac{{\hat{\eta }}_{N}}{{\hat{\sigma }}_{N}^{2}}$
1	2	23.33
	3	14.56
	4	13.72
	5	10.56
	6	6.09
2	2	50
	3	14.13
	4	13.33
	5	10.67
	6	6.73
3	2	16.25
	3	9.29
	4	7.66
	5	5.4
	6	4.88
4	2	62.5
	3	33.25
	4	25.45
	5	10.79
	6	8
5	2	22.22
	3	15.71
	4	9.68
	5	5.4
	6	4.25

**Table 7:** Computational time comparison for different process models.

Wiener process model		
Sample	Computation time per sample (s)	
1	0.0246	
2	0.0245	
3	0.0246	
4	0.0246	
5	0.0247	
IG process model		
Sample	Computation time per sample (s)	
1	0.0251	
2	0.0251	
3	0.025	
4	0.0252	
5	0.0251	
Two-phase hybrid stochastic degradation process model		
Sample	${\hat{q}}_{N}$	Computation time per sample (s)
1	2	0.569
	3	0.5692
	4	0.569
	5	0.5689
	6	0.569
2	2	0.569
	3	0.5692
	4	0.569
	5	0.569
	6	0.5691
3	2	0.569
	3	0.569
	4	0.569
	5	0.5691
	6	0.569
4	2	0.5691
	3	0.5691
	4	0.569
	5	0.5692
	6	0.5692
5	2	0.569
	3	0.569
	4	0.569
	5	0.569
	6	0.569

**Table 8:** K-S test results of the Wiener process model.

Wiener Process Model			
Critical Value $D_{40,0.05}^{Wiener}$	Statistic Value $D_{40}^{Wiener}$	*p*-Value	$D_{40}^{Wiener}<D_{40,0.05}^{Wiener},$ *p*-Value > *p*_original_
0.210	0.101	0.769	0.101<0.210,0.769>0.05

**Table 9:** K-S test results of the IG process model.

IG Process Model			
Critical Value $D_{40,0.05}^{IG}$	Statistic Value $D_{40}^{IG}$	*p*-Value	$D_{40}^{IG}<D_{40,0.05}^{IG},$ *p*-Value < *p*_original_
0.210	0.775	0	0.775>0.210,0<0.05

**Table 10:** K-S test results of the two-phase hybrid stochastic degradation process model.

First phase: Wiener process model			
Critical Value $D_{30,0.05}^{1}$	Statistic Value $D_{30}^{1}$	*p*-value	$D_{30}^{1}<D_{30,0.05}^{1},$ *p*-value > *p*_original_
0.242	0.119	0.736	0.119<0.242,0.736>0.05
Second phase: IG process model			
Critical Value $D_{10,0.05}^{1}$	Statistic Value $D_{10}^{1}$	*p*-value	$D_{10}^{1}<D_{10,0.05},$ *p*-value > *p*_original_
0.409	0.337	0.162	0.337<0.409,0.162>0.05

**Table 11:** The time to actual failure of the Wiener, IG, and two-phase hybrid process models.

Model	$\mathrm{Operating}\;\mathrm{Time}\;(h)\;\mathrm{When}\;R\left(t\right)=0.01$
Wiener	11,471
IG	7829
Two-phase	8684.5

## Data Availability

The data used in this paper are confidential.

## Appendix A

### Appendix A.1. Auto-Correlation Analysis of Degradation Increments

This appendix provides a complete calculation procedure based on the auto-correlation function (ACF) to analyze the auto-correlation characteristics of incremental performance degradation data. While Table 1 provides the calculation results, detailed step descriptions, all formulas, and the physical principles behind each feature are included below.

### Appendix A.2. Definition of Auto-Correlation Function

Suppose that the sequence of performance degradation increments obtained by the n-th test product over a given time interval is

(A1) $$\Delta X=\left\{\Delta x_{1},\Delta x_{2},\cdots ,\Delta x_{n}\right\}$$

The mean of the sequence is

(A2) $$\nu =\frac{1}{n}\sum_{\tau =1}^{n}\Delta x_{\tau }$$

where $\Delta x_{\tau }$ is the increment of the τ-th time interval.

The sample auto-covariance with lag order k is

(A3) $$\gamma_{k}=\frac{1}{n}\sum_{\tau =k+1}^{n}\left(\Delta x_{\tau }-\nu \right)\left(\Delta x_{\tau -k}-\nu \right)$$

where k=0,1,2,⋯ is the lag order. When k=0, $\gamma_{0}$ is the sample variance of the sequence.

The ACF of a sample with lag order k is

(A4) $$\theta_{k}=\frac{\gamma_{k}}{\gamma_{0}}=\frac{\sum_{\tau =k+1}^{n}\left(\Delta x_{\tau }-\nu \right)\left(\Delta x_{\tau -k}-\nu \right)}{\sum_{\tau =1}^{n}{\left(\Delta x_{\tau }-\nu \right)}^{2}}$$

The lag order k represents the degree of correlation between the increment $\Delta x_{\tau }$ in the current time interval and the increment $\Delta x_{\tau -k}$ in the previous k time intervals. Auto-correlation analysis is only performed for k=1.

### Appendix A.3. Significance Test of Auto-Correlation Function

Based on these steps, we establish the null hypothesis, $H_{0}:\theta_{k}=0,p_{k}>0.05$, and the alternative hypothesis, $H_{1}:\theta_{k}\neq 0,p_{k}\leq 0.05$. When the sequence does not exhibit significant non-stationarity, the sample ACF $\theta_{k}$ can be approximated by a normal distribution, and its statistic can be expressed as

(A5) $$J_{k}=\theta_{k}\sqrt{n},J_{k}\sim N\left(0,1\right)$$

The *p*-value for the significance test is

(A6) $$p_{k}=2\left[1-\psi \left(\left|J_{k}\right|\right)\right]$$

where $\psi \left(\cdot \right)$ represents the cumulative distribution function of the standard normal distribution. When $p_{k}$ is greater than the given significance level $p_{0}=0.05$, the null hypothesis $H_{0}$ cannot be rejected.

The results of the auto-correlation analysis are shown in Table A1. Although the sample auto-correlation coefficients $\theta_{1}$ are not numerically equal to 0, their corresponding p-values are all greater than 0.05, and their ranges are all within the 95% confidence interval of the null hypothesis $\frac{\pm 1.96}{\sqrt{8}}=\pm 0.693$. Therefore, the null hypothesis $H_{0}$ cannot be rejected.

**Table A1:** The results of the auto-correlation analysis (k=1).

Sample	ACF Value *θ*_1_	*p*-Value *p*_1_
1	0.572	0.106
2	0.586	0.098
3	0.588	0.096
4	0.627	0.076
5	0.628	0.076

## Appendix B. Derivation of the Formula for the Conditional Probability Density Function (PDF) $f_{X\left(t_{q}\right)}\left(x_{q}|x_{q}<\omega \right)$

Because the first phase is a Wiener process, the degradation status at the change point follows a normal distribution:

(A7) $$X\left(t_{q}\right)\sim N\left(\eta^{*}t_{q}{,\left(\sigma^{2}\right)}^{*}t_{q}\right)$$

As such, its unconditional PDF is

(A8) $$f_{X\left(t_{q}\right)}\left(x_{q}\right)=\frac{1}{\sqrt{2\pi {\left(\sigma^{2}\right)}^{*}t_{q}}}exp\left(-\frac{{\left(x_{q}-\eta^{*}t_{q}\right)}^{2}}{2{\left(\sigma^{2}\right)}^{*}t_{q}}\right)$$

The first phase of failure is defined as the degradation process that first reaches threshold ω, i.e.,

(A9) $$T_{1}=inf\left\{t|X\left(t\right)\geq \omega \right\}$$

Therefore, if the product does not fail before the change point $t_{q}$, the FPT in the range of $0\;{\leq t\leq t}_{q}$ is less than ω, i.e., $\left\{T_{1}>t_{q}\right\}$

(A10) $$f_{X\left(t_{q}\right)}\left(x_{q}|x_{q}<\omega \right)=\frac{P\left(X\left(t_{q}\right)\in dx_{q},T_{1}>t_{q}\right)}{P\left(T_{1}>t_{q}\right)},0<x_{q}<\omega$$

where $P\left(X\left(t_{q}\right)\in dx_{q},T_{1}>t_{q}\right)$ represents the probability that the degradation amount $X\left(t_{q}\right)$ at change point time $t_{q}$ is near $x_{q}$, assuming the product has not yet failed.

From Equation (36), we know that

(A11) $$P\left(T_{1}>t\right)=P\left(T_{1}>t_{q}\right)=R^{1}\left(t\right)=R^{Wiener}\left(t_{q}\right)$$

According to the reflection principle in Brownian motion [43], for any sample path that reaches or exceeds the threshold ω at least once within the time interval $0\;{\leq t\leq t}_{q}$ and returns to $x_{q}$ at time $t_{q}$, it can be mapped to a mirror path with its endpoint located at $2\omega -x_{q}$ under the condition of no boundaries. As the Wiener process has a constant drift term $\eta^{*}$, the mirror transformation will introduce a constant weight $exp\left(\frac{2\eta^{*}\omega }{{\left(\sigma^{2}\right)}^{*}}\right)$ containing the diffusion term $\sigma^{*}$ in the probability measure. Therefore, the expression for $P\left(X\left(t_{q}\right)\in dx_{q},T_{1}>t_{q}\right)$ can be obtained as follows:

(A12) $$P\left(X\left(t_{q}\right)\in dx_{q},T_{1}>t_{q}\right)=f_{X\left(t_{q}\right)}\left(x_{q}\right)-exp\left(\frac{2\eta^{*}\omega }{{\left(\sigma^{2}\right)}^{*}}\right)f_{X\left(t_{q}\right)}\left(2\omega -x_{q}\right)$$

Substituting Equation (A8) into Equation (A12), we obtain

(A13) $$P\left(X\left(t_{q}\right)\in dx_{q},T_{1}>t_{q}\right)=\frac{1}{\sqrt{2\pi {\left(\sigma^{2}\right)}^{*}t_{q}}}\cdot \left[exp\left(-\frac{{\left(x_{q}-\eta^{*}t_{q}\right)}^{2}}{2{\left(\sigma^{2}\right)}^{*}t_{q}}\right)\right]\;-exp\left(\frac{2\eta^{*}\omega }{{\left(\sigma^{2}\right)}^{*}}\right)exp\left(-\frac{{\left(2\omega -x_{q}-\eta^{*}t_{q}\right)}^{2}}{2{\left(\sigma^{2}\right)}^{*}t_{q}}\right)$$

We can see that ${\left(2\omega -x_{q}-\eta^{*}t_{q}\right)}^{2}={\left(x_{q}+\eta^{*}t_{q}-2\omega \right)}^{2}$.

Therefore, Equation (A13) can be rewritten as

(A14) $$P\left(X\left(t_{q}\right)\in dx_{q},T_{1}>t_{q}\right)=\frac{1}{\sqrt{2\pi {\left(\sigma^{2}\right)}^{*}t_{q}}}\cdot \left[exp\left(-\frac{{\left(x_{q}-\eta^{*}t_{q}\right)}^{2}}{2{\left(\sigma^{2}\right)}^{*}t_{q}}\right)\right]\;-exp\left(\frac{2\eta^{*}\omega }{{\left(\sigma^{2}\right)}^{*}}\right)exp\left(-\frac{{\left(x_{q}+\eta^{*}t_{q}-2\omega \right)}^{2}}{2{\left(\sigma^{2}\right)}^{*}t_{q}}\right)$$

By substituting Equations (A11) and (A13) into Equation (A10), we can obtain the expression for the conditional probability density, as shown in Equation (A15).

(A15) $$f_{X\left(t_{q}\right)}\left(x_{q}|x_{q}<\omega \right)=\frac{P\left(X\left(t_{q}\right)\in dx_{q},T_{1}>t_{q}\right)}{R^{Wiener}\left(t_{q}\right)}\;=\frac{1}{\sqrt{2\pi {\left(\sigma^{2}\right)}^{*}t_{q}}}\cdot \frac{exp\left(-\frac{{\left(x_{q}-\eta^{*}t_{q}\right)}^{2}}{2{\left(\sigma^{2}\right)}^{*}t_{q}}\right)-exp\left(\frac{2\eta^{*}\omega }{{\left(\sigma^{2}\right)}^{*}}\right)exp\left(-\frac{{\left(x_{q}+\eta^{*}t_{q}-2\omega \right)}^{2}}{2{\left(\sigma^{2}\right)}^{*}t_{q}}\right)}{R^{Wiener}\left(t_{q}\right)}$$
